# Systemic Interleukins’ Profile in Early and Advanced Colorectal Cancer

**DOI:** 10.3390/ijms23010124

**Published:** 2021-12-23

**Authors:** Paulina Czajka-Francuz, Sylwia Cisoń-Jurek, Aleksander Czajka, Maciej Kozaczka, Jerzy Wojnar, Jerzy Chudek, Tomasz Francuz

**Affiliations:** 1 Department of Internal Medicine and Oncological Chemotherapy, Faculty of Medical Sciences in Katowice, Medical University of Silesia, 40-027 Katowice, Poland; sylwiacison@o2.pl (S.C.-J.); jwojnar@sum.edu.pl (J.W.); chj@poczta.fm (J.C.); tfrancuz@mp.pl (T.F.); 2 Department of General Surgery, Vascular Surgery, Angiology and Phlebology, Faculty of Medical Sciences in Katowice, Medical University of Silesia, 40-635 Katowice, Poland; optok@wp.pl; 3 Department of Radiotherapy and Chemotherapy, National Institute of Oncology, Public Research Institute in Gliwice, 44-101 Gliwice, Poland; kozaczkam@interia.pl; 4 Department of Biochemistry, Faculty of Medical Sciences in Katowice, Medical University of Silesia, 40-752 Katowice, Poland

**Keywords:** tumor microenvironment, colorectal cancer, cytokine, inflammation

## Abstract

Tumor microenvironment (TME) is characterized by mutual interactions of the tumor, stromal and immune cells. Early and advanced colorectal tumors differ in structure and present altered serum cytokine levels. Mutual crosstalk among TME infiltrating cells may shift the balance into immune suppressive or pro-inflammatory, antitumor response this way influencing patients’ prognosis. Cancer-related inflammation affects all the body and this way, the systemic level of cytokines could reflect TME processes. Despite numerous studies, it is still not known how systemic cytokines levels change during colorectal cancer (CRC) tumor development. Better understanding tumor microenvironment processes could help in planning therapeutic interventions and more accurate patient prognosis. To contribute to the comprehension of these processes within TME, we reviewed cytokines levels from clinical trials in early and advanced colorectal cancer. Presented data were analyzed in the context of experimental studies and studies analyzing tumor infiltration with immune cells. The review summarizes clinical data of cytokines secreted by tumor microenvironment cells: lymphocytes T helper 1 (Th1), lymphocytes T helper 2 (Th2), lymphocytes T helper 17 (Th17), regulatory T cells (Treg cells), regulatory T cells (Breg cells), M1/M2 macrophages, N1/N2 neutrophils, myeloid-derived suppressor cells (MDSC), dendritic cells (DC), innate lymphoid cells (ILC) natural killer (NK) cells and tumor cells.

## 1. Introduction

An increasing body of evidence supports the crucial role of cytokines in the development of colorectal cancer (CRC). Cytokines can suppress tumor growth via pro-inflammatory action or contribute to tumor progression via immunosuppression, angiogenesis, or facilitation of immune escape [1].

Patients in various stages of colon cancer are characterized by different cytokine serum profiles [2]. Defining serum biomarker profiles characteristic in the subsequent stages of CRC may improve diagnosis of the early-stage disease, recurrence, or progression, and by this way contribute to reductions in mortality [3]. Understanding of stage-specific cytokine profile could provide further insights into the tumor microenvironment (TME) targeted therapeutic interventions. For example, the plasticity of pro-tumor or antitumor polarization lymphocytes T helper 1 (Th1), lymphocytes T helper 2 (Th2 cells), M1 and M2 macrophages, N1 and N2 neutrophils) gives the possibility of reprogramming of immunosuppressive TME and turning “cold” tumors into “hot” via TME targeting drugs [4]. Compartmentation of the immune response into inflamed, immune-excluded, and immune-desert phenotypes has been proposed as the major predictor of response to cancer treatments [5,6].

Still, there is a paucity of data regarding cytokines profile changes during the development of CRC as well as about mechanisms within the tumor leading to changes in the cytokine levels. The current review aimed to summarize available clinical data of chosen cytokines levels in early and advanced CRC stages. Data were analyzed in the context of experimental studies and studies showing infiltration of tumors with immune cells. The review presents data of cytokines secreted by lymphocytes Th1, Th2, Th17, regulatory T cells (Treg cells), regulatory B cells (Breg cells) as well as M1/M2 macrophages, N1/N2 neutrophils, myeloid-derived suppressor cells (MDSC), dendritic cells (DC), innate lymphoid cells (ILC) natural killer (NK) cells and tumor cells. The data were presented in a non-quantitative, descriptive manner. Systemic cytokine levels were presented according to clinical stages reported in original articles. Source papers reported CRC stages according to American Joint Committee on Cancer system, assessing tumor, nodes, and metastasis (TNM) in CRC patients. Shortly, stage 0 was considered as the earliest phase—carcinoma in situ, with the tumor not grown beyond the mucosa of the colon or rectum. In stage I bigger tumor could invade muscularis propria and in stage II tumor may infiltrate adjacent organs, however, in stages 0–II there is no lymph nodes involvement or distant metastasis found. In stage III, additionally, lymph nodes are infiltrated with the cancer cells, but still, there are no distant metastasis. Stage IV patients suffer from advanced disease with metastasis present in distant organs.

## 2. Review

**Figure 1:**
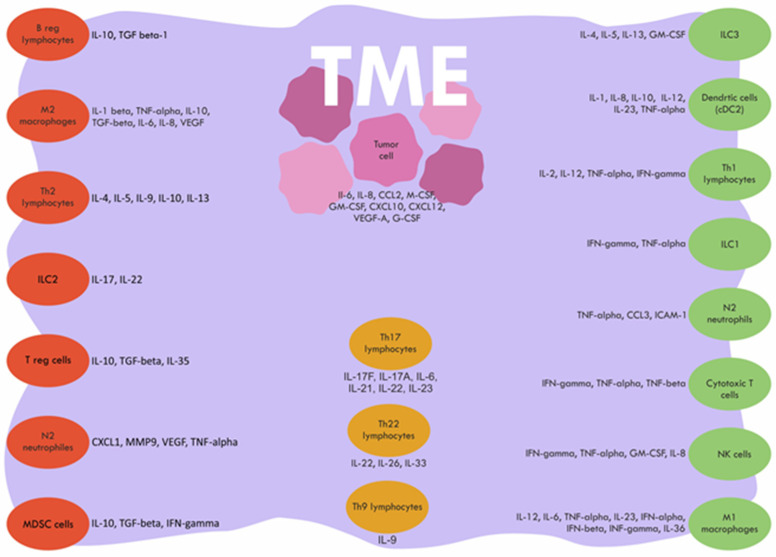
Several cell types in tumor histology were found to be associated with poor patient outcomes. These cells include M2 tumor-associated macrophages, N2 neutrophils, MDSCs (myeloid-derived suppressor cells), regulatory T cells (Treg cells), subsets of T helper lymphocytes: Th2, Th9, and Th17 cells, cancer-associated fibroblasts (CAF), and regulatory B cells (Breg) cells playing mostly tumor-promoting functions. Other cells in TME showed antitumor activity related to a favorable prognosis. This group of cells includes tumor associated macrophages of M1 phenotype, N1 neutrophils, Th1 cells, cytotoxic T cells, innate lymphoid cells (ILC) with natural killer (NK cells) cells. Some cells subtypes, like Th9, Th17, Th22 may play a dual, pro-tumor, or antitumor role depending on the TME polarization. Mutual crosstalk among TME cells may shift the balance into immune suppressive or antitumor immunity, this way influencing patients’ prognosis.

The tumor microenvironment plays a central role in the immune response against tumors in cancer, including CRC. From the early stages of CRC, cytokines can be produced by tumor-infiltrating immune cells, stromal cells, and tumor cells (Figure 1). If we look at early tumor structure, there are small tumors with many CD3, CD8, CD4, memory T cells, and tertiary lymphoid organs (TLO) at the invasive margin and in the tumor center. T2 and T3 tumors are larger and progressively have fewer T lymphocytes, fewer lymphatic vessels, and more angiogenesis [7,8]. Moreover, smaller tumors do not show significant hypoxia, which promotes macrophage polarization towards the M2 phenotype and modifies the microenvironment by decreasing the release of antitumor cytokines [9]. The structure of advanced, T4 tumors differs from earlier stages. There are very few T lymphocytes present, more intensive angiogenesis, and domination of macrophages in advanced CRC stages. Tumor-associated macrophages (TAMs) constitute up to 50% of the tumor mass cells in the TME of advanced CRC [10,11]. Lymphocytes and neutrophils are also observed, but few plasma cells or eosinophils are present [12,13,14]. Additionally, tumor cells themselves produce immunosuppressive cytokines such as interleukin 6 (IL-6), vascular endothelial growth factor (VEGF), transforming growth factor-beta (TGF-beta), and others, and can convert effector T cells into regulatory T cells, this way contributing to immune-suppressive TME. This influence might be more important in the later stages, due to the higher number of tumor cells [15].

Still, it is not fully known, how the tumor structure translates into cancer-related inflammation and systemic cytokine levels. To address this question, we analyzed cytokines levels from clinical trials in different CRC stages. Results are shown in Table 1 and Table 2 and discussed in the following sections.

**Table 1:** Systemic cytokine levels in CRC patients vs. controls. Groups containing more than 1 stage were assessed as a whole.

Cytokine	1 vs. Control	II vs. Control	III vs. Control	I-III vs. Control	II/III vs. Control	0-IV vs. Control; Stage 0—In Situ Cancer	I-IV vs. Control	II-IV vs. Control	IV vs. Control
IL-1RA				Not increased [16]	Not increased [17]	Not increased [18]	Increased [19]	Not increased [17]	Not increased [17]
IL-1 beta	Increased [20]	Increased [20]	Increased [20]	Increased [16]	Increased [17]	Not increased [18]	Increased [20,21]	Increased [17]	Increased [17,20]
IL-2				Not increased [16]	Not increased [17,22]			Increased [17]	Not increased [17]
IL-4				Not increased [16]	Increased [17] Not increased [22]	Increased [18]		Increased [17]	Increased [17]
IL-5				Not increased [16]	Increased [17]	Not increased [18]		Increased [17]	Not increased [17]
IL-6	Increased [20]	Increased [20]	Increased [20]	Not increased [16,23]	Not increased [22] Increased [17]	Not increased [18]	Increased [19,20,21]	Increased [17]	Increased [17,20,24]
IL-7				Increased [16]	Not increased [17]	Not increased [18] Increased [25]		Not increased [17]	Not increased [17]
IL-8	Not increased [20]	Increased [20]	Increased [20]	Increased [16]	Increased [17,22]	Increased [18]	Increased [19,20,26,27]	Increased [17]	Increased [17,20]
IL-9				Not increased [16]	Increased [17]	Increased [18]	Decreased [28]	Not increased [17]	Not increased [17]
IL-10	Increased [29]	Increased [29]	Increased [29]	Not increased [16]	Not increased [17,22]	Not increased [18]	Not increased [19] Increase [29,30]	Not increased [17]	Not increased [17] Increase [29]
IL-12	Increased [31]	Increased [31]	Increased [31]	Not increased [16]	Not increased (ja) [17]	Not increased [18]	Increase [31]	Not increased [17]	Not increased [17]
IL-13				Not increased [16]	Not increased [17]	Not increased [18]		Not increased [17]	Not increased [17]
IL-15				Not increased [16]					
IL-17A				Not increased [16]	Not increased [17,22]	Increased [18]	Increased [21,32]	Not increased [17]	Not increased [17]
IL-23	Increased [29]	Increased [29]	Increased [29]				Increased [21,29]		Increased [29]
IFN-gamma				Not increased [16]	Not increased [22]	Increased [18]			
TNF-alpha	Increased [20]	Increased [20]	Increased [20]	Increased [16]		Increased [18]	Increased [19,20]		Increased [20]
TGF -beta		Increased [33]	Increased [33]						
G-CSF	Not increased [20]	Increased [20]	Increased [20]	Increased [16]	Increased [22]	Increased [18]	Increased [20]		Increased [20]
GM-CSF	Increased [20]	Increased [20]	Increased [20]	Not increased [16]	Not increased [22]	Not increased [18]	Increased 20]		Increased [20]
M-CSF							Increased [19]		
VEGF				Increased [23] Not increased [16]	Increased [22]	Not increased [18]			
VEGF-A	Increased [20,34]	Increased [20,34]	Increased [20,34]				Increased [20]		Increased [20,34]
VEGF-B	Increased [34]	Increased [34]	Increased [34]						Increased [34]
VEGF-C	Not increased [34]	Increased [34]	Increased [34]						Increased [34]
VEGF-D	Not increased [34]	Not increased [34]	Increased [34]						Increased [34]

Abbreviations: IL-1RA—interleukin 1 receptor antagonist; IL-1 beta—interleukin 1 beta; IL-2—interleukin 2; IL-4—interleukin 4; IL-5—interleukin 5; IL-6—interleukin 6; IL-7—interleukin 7; IL-8—interleukin 8; IL-9—interleukin 9; IL-10—interleukin 10; IL-12—interleukin 12; IL-13—interleukin 13; IL-15—interleukin 15; IL-17A—interleukin 17A; IL-23—interleukin 23; IFN-gamma—interferon gamma; TNF-alpha—tumor necrosis factor alpha; TGF-beta—transforming growth factor-beta; G-CSF—granulocyte colony-stimulating factor; GM-CSF—granulocyte-macrophage colony-stimulating factor; M-CSF—macrophage colony-stimulating factor; VEGF—vascular endothelial growth factor; VEGF- A—vascular endothelial growth factor A; VEGF-B—vascular endothelial growth factor B; VEGF-C—vascular endothelial growth factor C; VEGF-D—vascular endothelial growth factor D.

**Table 2:** Systemic cytokine levels in patients with different stages of CRC.

Cytokine	Stage IV vs. I	Stage IV vs. II	Stage IV vs. III	Stages III, IV vs. I/II	Stages IV vs. I-III
IL-1 beta	Increased [20]	Not increased [20]	Not increased [20]	Increase [21]	
IL-6	Increased [20]	Not increased [20]	Not increased [20]	Not increased [21]	Increased [24,35]
IL-8	Increased [20]	Not increased [20]	Not increased [20]		Increased [26,27,35]
IL-9				Decrease [28]	
IL-10	Increased [29]	Increased [29]	Increased [29]	Increased [30]	Increased [30]
IL-12	Decreased [31]	Decreased [31]	Decreased [31]		
IL-17A				Increased [21,32]	
IL-17F					Increased [36]
IL-23				Increased [21]	Increased [36]
IFN-gamma					Increased [36]
TNF-alpha	Not increased [20]	Not increased [20]	Not increased [20]		Not increased [36]
G-CSF	Increased [20]	Not increased [20]	Not increased [20]		
GM-CSF	Not increased [20]	Not increased [20]	Not increased [20]		
VEGF					Increased [24]
VEGF-A	Increased [20,34]	Not increased [20,34]	Not increased [20,34]		
VEGF-B	Not increased [34]	Not increased [34]	Decreased [34]		
VEGF-C	Increased [34]	Not increased [34]	Not increased [34]		
VEGF-D	Increased [34]	Increased [34]	Not increased [34]		

Abbreviations: IL-1 beta—interleukin 1 beta; IL-6—interleukin 6; IL-8—interleukin 8; IL-9—interleukin 9; IL-10—interleukin 10; IL-12—interleukin 12; IL-17A—interleukin 17A; IL-17F—interleukin 17F; IL-23—interleukin 23; IFN-gamma—interferon gamma; TNF-alpha—tumor necrosis factor alpha; G-CSF—granulocyte colony-stimulating factor; GM-CSF—granulocyte-macrophage colony-stimulating factor; VEGF—vascular endothelial growth factor; VEGF-A—vascular endothelial growth factor A; VEGF-B—vascular endothelial growth factor B; VEGF-C—vascular endothelial growth factor C; VEGF-D—vascular endothelial growth factor D.

### 2.1. Immunosuppressive Cells/Tumor Cells and Their Cytokines Panel

#### 2.1.1. Lymphocytes Th2

Th2 cells show mainly immunosuppressive action supporting tumor development and act opposite to Th1 lymphocytes which contribute to anticancer immunity. Th2 lymphocytes stimulate macrophage differentiation into pro-tumor M2 phenotype in TME, maintain the differentiation of Th2 naïve lymphocytes into Th2 cells and promote humoral responses via stimulating B cells. Th lymphocytes demonstrate some level of plasticity, such that Th1 cells can differentiate into Th17, Th2, and Treg cells upon chronic stimulation in suppressive TME [37]. Lymphocytes Th2 secrete interleukin 4 (IL-4), interleukin 5 (IL-5), interleukin 9 (IL-9), interleukin 10 (IL-10), interleukin 13 (IL-13) [38]. Basophils, mast cells, and innate lymphoid cells type 2 (ILC2) cells are activated by Th2 cytokines and share a similar cytokines secretion profile to Th2 cells.

CRC tumor microenvironment is described by a shift from Th1 to Th2 cytokines in the blood and increased immunosuppression within TME. Indeed, in analyzed studies, it was possible to observe several Th2 cytokines increase in early CRC stages, namely IL-4 and IL-5 (clinical stages II and III) vs. control. Moreover, IL-10 level was elevated in CRC patients staged I, II, and III vs. control. In advanced CRC (stage IV patients) a shift into Th2 profile was demonstrated, with the increased level of IL-4 and IL-10. Taking into account tumor organization, we hypothesize that increased levels of IL-4 and IL-5 in earlier CRC stages (I-III) could be a result of production by Th2 cells rather than ILC2 cells. We presume, that IL-10 at early CRC stages is produced by Th2 cells rather than M2 macrophages, which secrete high amounts of IL-10 but are not numerous in early disease [39].

##### IL-4

A shift into Th2 cells in tumors was associated with a worse prognosis in CRC, which was contributed to IL-4 release [40]. This cytokine promotes epithelial to mesenchymal transition (EMT) [41], tumor cell proliferation, invasion, and metastasis in CRC [42,43]. Upregulation of IL-4 was found to be a mechanism protecting the tumorigenic CD133+ cells from apoptosis in an animal model [44]. Moreover, it was demonstrated that IL-4 increased production of reactive oxygen species (ROS) in CRC, contributed to tumor mediated inflammation and tumor progression [38]. Some authors proposed a mechanism of immune escape for tumor-initiating cells related to IL-4 [45]. During tumor development increased expression of interleukin-4 receptor (IL-4R) and elevated IL-4 levels were found in CRC [46]. Experimental data are translated into the clinical picture, as higher serum levels of IL-4 were found in CRC patients with metastases (M1) compared with patients without metastases (M0) [2].

On the contrary, IL-4 has also been shown to inhibit tumor growth and progression in other tissues, such as kidney cancer [47], which was dependent on tumor-specific CD8+ T cells. Moreover, Th2 immune responses induced IL-4 and eosinophil-dependent anti-tumor activity [48]. So IL-4 may have distinct functions, pro- and antitumor, depending on the tumor environment. However, based on the published evidence, IL-4 appears to support CRC development.

Th2 lymphocytes are not the only source of IL-4 in CRC patients. It was demonstrated that also ILC2, double-positive CD4+ CD8+ T cells, and cancer-initiating cells secrete this cytokine [45,49]. Due to the relatively low number of tumor cells in early CRC stages as well as ILC2 cells, the Th2 cells seem to be the main source of IL-4 in the II and III stages.

##### IL-13

IL-13 is closely related to IL-4 and is secreted also by ILC2. Both IL-4 and IL-13 act via interleukin-4 receptor alpha (IL-4R alpha). Sharing a common receptor limits a clear differentiation of IL-4 and IL-13 action in CRC. Although the level of IL-13 was not different from controls in analyzed studies (Table 1), experimental data suggest that IL-13 signaling could be involved early in intestinal stem cell self-renewal and homeostasis [50]. IL-13 cells could promote intestinal stem cell renewal via activation of signal transducer and activator of transcription 6 (STAT6) and Foxp1-dependent pathways in crypt intestinal stem cells. Moreover, IL-13 can activate MDSC cells this way promoting a tumorigenic microenvironment [51]. On the other hand, through the secretion of IL-13, ILC2 has been shown to promote migration of dendritic cells and activation of cytotoxic T cells, which might support antitumor immunity [52]. IL-13 role in CRC TME needs further research and explanation.

##### IL-5

IL-5 seems to show mainly antitumor properties in CRC. IL-5 was found elevated in early CRC stages (Table 1). It was demonstrated that IL-5 may stimulate eosinophils in TME [53]. Some authors reported a high eosinophils infiltration score in the tumor to be negatively correlated with patient age and tumor stage [54]. Others showed that increased peri- and intratumoral eosinophil counts were associated with T and N classification, tumor differentiation, and vascular invasion [55]. It appears that in CRC patients, the presence of eosinophils in the tumor center may influence the activation of the immune system and predict a better prognosis [56]. IL-5 can be secreted by Th2 and ILC2 cells in TME. However, due to the low number of ILC2 cells in early CRC stages [57], Th2 cells could be considered as the main source of IL-5 in non-metastatic CRC.

##### IL-9

Another Th2 cell cytokine, IL-9, was elevated in patients staged 0-IV vs. control (Table 1), however, other reports showed decreased level of IL-9 in group staged I-IV CRC compared to controls [28]. Moreover, IL-9 level was decreased in patients in stages III-IV vs. I-II, suggesting a more pronounced role in the early CRC stages. Several studies suggest a dual role of IL-9 in CRC pathogenesis. This regulatory cytokine is secreted by Th2 cells, lymphocytes T helper 9 (Th9), Th17, and Treg cells. IL-9 enhances the expansion of cytotoxic T cells inhibiting CRC development by binding to interleukin 9 receptor (IL-9R) expressed on CD8+ T cells [58]. A recent study also confirmed the antitumor effect of IL-9, demonstrating that overexpression of IL-9 inhibited tumor growth in vivo [59]. IL-9 is known for promoting the proliferation and growth of mast cells, present in the mucous membrane of the colon, which might protect against cancer development in the early stages [60]. In CRC, it was demonstrated that the main source of IL-9 was mainly Th9 cells [61]. However, the role of IL-9 has to be assessed more precisely in future studies.

##### IL-10

In TME IL-10 is mainly produced by M2 macrophages and Th2 cells but also monocytes, mast cells, Treg cells, and by subsets of activated T and B cells [62]. This cytokine plays a primarily immune suppressive role in TME. IL-10 inhibits the synthesis of the pro-inflammatory cytokines interleukin 1 (IL-1), interleukin 12 (IL-12), tumor necrosis factor-alpha (TNF-alpha), and interferon-gamma (IFN-gamma) by stimulated monocytes/macrophages [63]. IL-10 can control T cell responses including IFN-gamma secretion and blocking proliferation of T cells [64,65]. Moreover, IL-10 downregulates the secretion of antitumor Th1 cytokines, the expression MHC class II antigens, and co-stimulatory molecules on macrophages [66]. IL-10 promotes the differentiation of B cells into plasma cells this way supporting immune suppression [67]. IL-10 acts by initiating the signaling via signal transducer and activator of transcription protein 3 (STAT3) pathway [68]. High preoperative serum levels of IL-10 correlated with poor prognosis in CRC patients [69] and patients with cancer recurrence after surgery had a significantly higher level of IL-10, indicating that IL-10 could be considered as the prognostic biomarker in CRC [30].

Immune suppressive and tumor-promoting action of IL-10 was supported by finding that IL-10 deficiency enhanced the efficacy of DC-based immunotherapy, reduced MDSC and Treg levels in the TME, and promoted Th1-type antitumor responses in mice [70]. In contrast, in vitro studies have shown, that low levels of IL-10 could exert the antitumor effect via activation of NK cells, T lymphocytes, and macrophages [62]. These data show that the role of IL-10 still has to be fully explained.

Cytokine levels from clinical studies indicate, that in CRC patients serum IL-10 levels increase over time during cancer progression [71,72]. IL-10 level was found to increase in I, II, III, but also in stage IV compared to controls, suggesting a crucial role of this cytokine across all stages of the disease (Table 1). Taking into account tumor structure, we asume that an increased level of IL-10 in non-metastatic disease (stages I, II, III) could be predominantly a result of Th2 cells secretion. IL-10 level is also increasing across CRC stages (stages III/IV vs. I/II and stage IV vs. stages I, II, III) (Table 2). It might reflect increasing tumor mass (including metastases) and enhancement of immune-suppressive processes. Apart from Th2 cells, IL-10 is secreted also by M2 macrophages, ILC2 cells, Th9 cells, and Tregs [73]. With known M2 predominance in the advanced CRC stage, increased levels of IL-10 in stage IV could be a result of M2 macrophages secretion [74,75] but also MDSC and Treg cells production, as the number of these cells increases with the tumor stage. This concept would be supported by an increased level of other M2 cytokines–VEGF, VEGF-A, IL-6, and IL-8, which all were found increased in stage IV CRC patients compared to controls.

#### 2.1.2. Lymphocytes Th17 and Their Cytokine Panel

Th17 cells are considered a separate subset of CD4+ effector lymphocytes and are characterized by secretion of interleukin 17F (IL-17F), interleukin 17A (IL-17A), IL-6, interleukin 21 (IL-21), interleukin 22 (IL-22), interleukin 23 (IL-23) [73,76]. The importance of Th17 cells in CRC development was confirmed in a meta-analysis of randomized trials [77]. Compared with control subjects, CRC patients showed elevated levels of serum IL-17A, IL-6, IL-22, and IL-23 [77,78]. Other findings indicated that Th17 cells inhibit the recruitment of CD8+ T cells via IL-17A/STAT3 signaling [79]. Current evidence suggests a dual role of Th17 cells in CRC development and underlines the necessity of further works and analyses of this cells population [80].

##### IL-17A

The main source of IL-17A and IL-17F in TME are Th17 cells, but both cytokines are also produced by Tgamma delta lymphocytes, NKT cells, neutrophils, and eosinophils [81]. Pro-tumor action of IL-17A was confirmed by numerous studies. IL-17A has been shown to recruit MDSCs cells and decrease antitumor immunity [82]. Increased level of IL-17A was associated with increased production of VEGF and poor prognosis in CRC [83]. IL-17A enhanced tumor growth in vivo via induction of IL-6, activating oncogenic transcription factor STAT3 and stimulating pro-survival and pro-angiogenic tumor genes [84].

Mixed clinical data were reported for IL-17A levels in CRC (Table 1). In the majority of studies, IL-17A concentration was found elevated in all stages. Moreover, a higher level was reported in stages III/IV than I/II. This finding is supported by a higher percentage of Th17 cells in cancer tissues in patients with advanced stages than in those with early stages [21,85]. Another study showed Th17 cells number was growing in tumor stroma during the colorectal adenoma-carcinoma sequence [86]. It might suggest that IL-17A plays a more pronounced tumor-promoting role in more advanced stages of CRC. This hypothesis could be supported by a higher level of IL-17A associated with a more advanced CRC stage and the occurrence of metastasis [32,87]. Immunofluorescence assays analysis showed that IL-17A was predominately produced by CD4+ T cells rather than from CD8+ T cells. However, contradictory findings were also reported. Some authors described a significantly reduced number of Th17 cells in CRC tumors [88]. To summarize, an increase in IL-17A could be associated with an increasing number of Th17 cells in a more advanced CRC stage, but further studies are required to assess the source of this cytokine and the mechanisms of its action.

##### IL-17F

Contrary to IL-17A, IL-17F was supposed to possess antitumor activity in animal models [89]. IL-17F inhibited tumor angiogenesis by modulating VEGF levels [90]. However, it was found that IL-17F could act as an oncogene in CRC [91,92]. IL-17F was found elevated in stage IV CRC patients vs. stage I-III patients. Similar to IL-17A, an increase of IL-17F level could be associated with the increase of Th17 cells number observed with higher CRC stage, but further studies are required to assess precisely the source of this cytokine in CRC patients.

##### IL-23

Dual, pro-tumor, and antitumor action of IL-23 was reported so far. IL-23 demonstrated several antitumor properties in experimental studies [93]. IL-23 induced activated memory T cells to proliferate and produce antitumor IFN-gamma [94]. On the other hand, pro-tumor action of IL-23 was also observed, including increased production of interleukin 17 (IL-17) by IL-23 activated Th-17 cells [95]. IL-23 was also involved in the expansion and maintenance of pro-tumor memory Th17 cells [96]. IL-23 was known to promote the pro-inflammatory and regenerative activities of Th17 cells and innate lymphoid cells [97]. Based on published evidence it seems that pro-tumor action of IL-23 prevails in CRC.

Increasing values of IL-23 with cancer stages were observed [98]. IL-23 level was elevated in stages I, II, III, IV patients vs. control (Table 1). IL-23 was also increased when comparing stage III/IV vs. I/II patients as well as in stage IV patients when comparing to stages I, II, III (Table 2). Apart from Th17 cells, IL-23 can be also produced by activated dendritic cells, macrophages, and monocytes. Based on published evidence it could be presumed that in earlier stages of CRC the major source of IL-23 could be the Th17 cells, similarly to other Th17 cytokines. In more advanced stages, the growing number of macrophages M2 in the tumor could contribute to IL-23 secretion. However, based on currently reported data it is not possible to establish sources of IL-23 in CRC patients.

#### 2.1.3. M2 Macrophages and Their Cytokines Panel

Pro-tumor M2 macrophages are considered the most important cells contributing to immunosuppression within TME. Tumor-associated macrophages (TAMs) dominate in TME in primary operable CRC tumors [12,13,14,99]. M2 polarized macrophages promote immunosuppression and decrease cancer-related inflammation [100]. They are the most commonly found phenotype of macrophages within the tumor [39]. M2 macrophages recruit granulocytes, Th2, and regulatory T cells through the production of CCL17, CCL18, CCL22, and CCL24 chemokines [101,102]. Main cytokines secreted by M2 macrophages include interleukin 1 beta (IL-1 beta), TNF-alpha, IL-10, transforming growth factor-beta (TGF-beta), IL-6, IL-8, and VEGF.

##### IL-1 Beta

The role of IL-1 beta in CRC is not clear. Despite mainly pro-tumor properties, antitumor actions were also proved. Among pro-tumor actions, IL-1 beta promoted the recruitment of immunosuppressive MDSC cells to tumors, which supported cancer progression [103,104]. IL-1 beta showed to stimulate intestinal epithelial cells and tumor cells to induce their proliferation [105]. Blockade of IL-1 beta using recombinant IL-1RA significantly decreased tumor development in the mouse model of colitis-associated cancer, reinforcing the pro-tumorigenic role of IL-1 beta [106]. Moreover, IL-1 beta-induced the activation of the Wnt signaling pathway by phosphorylation of GSK3beta [105]. These signaling pathways are known to play a key role in intestinal tumorigenesis [105,107]. Moreover, IL-1 beta-induced expression of TNF-alpha, IL-6, IL-8, IL-17, COX-2, and PGE2, pro-inflammatory mediators, and pro-tumor factors supporting tumor growth cells [108]. On the contrary, antitumor properties were also reported. IL-1 strengthened antigen response in CD4 and CD8 T cells, inducing expansion and activation of Th1, Th2, and Th17 cells in mice [109]. IL-1 was necessary for naïve CD4+ T cells to overcome Treg-mediated inhibition and for memory CD4+ T cells to acquire a functional memory phenotype in mice [110]. Although IL-1 beta is an important component of Th17 cell differentiation, it could also promote other T-cell responses, including CD8+ cells [111]. Of note, IL-1 beta in combination with IL-23 enhanced plasticity of Th17 cells into Th1 phenotype [112]. IL-1 beta strengthened also Th9 cell function via the interferon regulatory factor 1-dependent increase in the production of IL-21, which in turn stimulated IFN-gamma production and antitumor activity of CD8+ and NK cells [113].

Increased IL-1 beta level was shown in each clinical stage (I, II, III, IV) than in controls. IL-1 beta level was elevated in stages III/IV compared to I/II stages, in the majority of analyzed studies (Table 1). Activated myeloid cells, macrophages, and monocytes are considered the main sources of IL-1 beta in TME [114]. In the early stages of carcinogenesis, IL-1 beta exhibited pro-inflammatory, tumor-invasion-promoting, and immunosuppressive activity [115,116]. We propose M2 macrophages to be a major source of IL-1 beta in CRC patients, as they consist of up to half of the tumor mass in advanced stages [8,9]. This could explain a clear increase in IL-1 beta level with tumor development. However further studies are required to explain better IL-1 beta’s role in CRC.

##### IL-8

IL-8 is one of the major players contributing to tumor growth. In addition to promoting angiogenesis, proliferation, invasion, migration, and survival of CRC cells, IL-8 and was shown to induce the epithelial-mesenchymal transition of colorectal cancer cells helping them to escape from host immune defense [117]. IL-8 was found to support resistance to anoikis in tumor cells, which promoted the formation of circulating tumor cells and metastasis formation [118]. High IL-8 levels were associated with increased neutrophil infiltration into tumors and poor responses to the immune-checkpoint blockade [119,120]. Moreover, an elevated level of IL-8 before treatment was correlated with the progressive disease [27,35].

IL-8 is mainly secreted by M2 macrophages and monocytes, but it could be also produced by endothelial cells under exposure to IL-1 or TNF-alpha. Additionally, fibroblasts and malignant tumor cells could also secrete IL-8 as a result of environmental stress including hypoxia, and chemotherapy agents [121], however, it was not confirmed for CRC cells.

When analyzing clinical data, IL-8 level was increased in stages I-III or I-IV vs. controls and in stage IV vs. stages I-III (Table 1 and Table 2). These data support a central role of IL-8 in the development and progression of CRC. This may suggest a pronounced role of IL-8 in angiogenesis and attracting immune suppressive cells in more advanced CRC stages. We hypothesize the main source of IL-8 in early and advanced tumors could be M2 macrophages, due to their high prevalence in tumor tissue from the beginning of tumor growth.

##### IL-6

Another M2 cytokine, IL-6, is considered one of the major players in CRC TME, showing mainly immunosuppressive properties. IL-6 is involved in the differentiation of monocytes to macrophages, increase apoptosis of cytotoxic T cells, and the production of Th2 cytokines [122]. IL-6 is considered a growth factor for colon cancer cells; inhibition of IL-6 signaling slowed down the tumor cell’s growth [123]. Through the production of IL-6, M2 macrophages could influence the EMT process, intensifying the migration of CRC cells [124]. It was demonstrated, that IL-6 contributed to the acceleration of tumor progression and increased migration of CRC cells [125]. Moreover, the presence of sIL-6R and IL-6 stimulated Th17 cells and was responsible for the balance between Th17 and Tregs [123]. IL-6 promoted durable and long-lived Th17-mediated antitumor immunity [126]. It was also shown, that the IL-6/STAT3 pathway blocked the maturation of dendritic cells, inhibited T cell activation [127], and maintained immunosuppression through MDSC and M2 macrophages attraction. A high level of IL-6 was associated with poor prognosis in CRC patients [128]. Apart from M2 macrophages, IL-6 could be also produced by endothelial cells, B cells, T cells, fibroblasts. Moreover, it was demonstrated that IL-6 can be secreted by human colorectal cancer cells, together with IL-1 beta, IL-6 receptors (IL-1R1 and IL-6R), and VEGF [129].

In analyzed studies, serum IL-6 level was increased in all stages of CRC compared to controls (Table 1). When comparing IL-6 levels between stages, the difference was significant when compared to stage I, only (Table 2). This observation may suggest an important role of IL-6 since the early stages of tumor development, maintained during further tumor growth and invasion. The number of M2 macrophages is relatively low at the early stages of CRC, so IL-6 could be produced by tumor cells and M2 macrophages, creating propel wheel for further tumor growth. At later stages, M2 macrophages might contribute to the greater extent to IL-6 secretion. However, this hypothesis requires verification in future studies.

##### VEGF

Vascular endothelial growth factor (VEGF) is a potent angiogenic factor. There are different isoforms of this protein: VEGF-A, VEGF-B, VEGF-C, and VEGF-D [130] and others [131]. It was shown, that VEGF-A, VEGF-B, VEGF-C, and VEGF-D expression is modulated during the adenoma-carcinoma sequence in CRC. VEGF-A is upregulated in adenomas and carcinomas and VEGF-D was found more abundant in normal tissues [130]. High preoperative VEGF plasma levels were associated with worse survival in CRC patients [132].

VEGF-A is involved in inducing endothelial cell proliferation, migration, proteolytic activity, stimulating microvascular leakage, and promoting angiogenesis. VEGF-A mediates its function through vascular endothelial growth factor receptor 1 (VEGFR-1) and vascular endothelial growth factor receptor 2 (VEGFR-2) receptors. VEGF-A can promote lymphangiogenesis indirectly via the recruitment of bone marrow-derived macrophages, which release lymphangiogenic growth factors VEGF-C and VEGF-D. This shows the importance of VEGF-A, not only for pathological hemangiogenesis but also for lymphangiogenesis [133]. Moreover, VEGF-A actions include increased migration and mitosis of endothelial cells, increased matrix metalloproteinase activity, and chemotactic action for macrophages and granulocytes [134].

VEGF-C and VEGF-D are both involved in lymphangiogenesis [135]. In tumors, VEGF-C and VEGF-D are overproduced and activate vascular endothelial growth factor receptor 3 (VEGFR-3), supporting lymphatic vessels growth within the tumor [136]. Lymphatic vessels created through tumor angiogenesis are larger than normal, promoting the transfer of tumor cells to lymph nodes and supporting the formation of metastasis [137]. VEGF-B does not promote angiogenesis [138], however, its indirect role in angiogenesis was proposed [139,140].

Hypoxia induces the production of VEGF in tumor cells through hypoxia-inducible factor-1 alpha (HIF-1 alpha) [141]. This transcriptional factor regulates several genes including VEGF promoting angiogenesis and metastasis. VEGF proteins released by tumor cells into the extracellular space bind to VEGF receptors on endothelial cells and promote local angiogenesis with the formation of tumor-associated microvessels [142].

Apart from M2 macrophages, also tumor cells, cancer associated fibroblasts (CAFs), platelets, and mast cells can produce VEGFs, although they are not primary sources of VEGF proteins [143].

Increased VEGF levels in stage I CRC patients support the crucial role of this factor in tumor growth in the early stages [144,145]. Based on tumor structure, we propose that in the early stages the main source of VEGF proteins could be M2 macrophages. Moreover, we hypothesize that increased levels of VEGF A, B, C, D levels in CRC patients staged III and IV could be a result of increased production by MDCS cells and M2 macrophages supporting increased angio- and lymphangiogenesis in the late CRC stage. Tumor cells could contribute to an elevated level of VEGF-A in more advanced CRC stages. Increased levels of VEGF-B in stage III and VEGF-D in stages III and IV [34] could indicate the importance of angio- and lymphangiogenesis processes in advanced disease, contributing to immune escape and formation of metastasis, as supported by recent literature reports.

##### TGF-Beta

TGF-beta can be secreted by macrophages, immune cells, and tumor cells [146,147] as well as CAFs [148] in TME. TGF-beta and its receptors: transforming growth factor beta receptor 1 (TGFBR1) and transforming growth factor beta receptor 2 (TGFBR2), are commonly expressed on epithelial cells [149]. The role of TGF-beta in cancer is determined by mutations in its signaling [150,151]. TGF-beta exerts a dual function during intestinal tumorigenesis. In early tumors, TGF-beta was shown to act as a potent tumor suppressor as demonstrated in human [152] and animal models [150,153]. However, in later stages, its pro-tumor action was observed. Although the mechanisms underlying the dual role of TGF-beta for CRC remain to be explained, it was demonstrated that activation of TGF-beta signaling in epithelial cells induced the expression of cell-cycle checkpoint genes leading to growth arrest [154]. On the other hand, inhibition of TGF-beta signaling prevented metastasis or further development in advanced tumors CRC [155]. TGF-beta was shown to impair immune cell responsiveness [156] and promote angiogenesis [157]. High TGF-beta levels in the primary tumor and serum correlated with poor survival of advanced CRC patients [158]. Calon et al. showed that activation of TGF-beta signaling in fibroblasts promoted the metastatic capabilities of intestinal tumor cells [159]. According to Mager et al. [46], TGF-beta could indirectly exert a pro-tumorigenic effect on CRC cells, via the stroma, as TGF-beta may promote interleukin 11 secretion by CAFs [159], activating STAT3 and this way stimulating the proliferation of tumor cells [160].

Increased level of TGF-beta was reported in patients stage II and III compared to controls, confirming pro-tumorigenic action of this protein secreted mainly by M2 macrophages, MDSC cells, and tumor cells.

#### 2.1.4. MDSC Cells and Their Cytokines Panel

Myeloid-derived suppressor cells are a heterogeneous population of immature myeloid cells in differentiation phases originating from the myeloid progenitor stage. MDSC cells exhibit a range of tumor-promoting functions [161], emerging as a potential therapeutic target [162]. In lung, breast, and colorectal cancer the abundance of MDSCs in the tumor has been correlated with advanced stage and decreased overall survival [163]. MDSC are derived from myeloblasts, which are precursors to neutrophils and myeloid-dendritic cell progenitors. These cells could differentiate into monocytes, but due to stimulation with tumor secreted factors do not mature and form MDSCs. MDSCs could be divided into monocytic MDSCs (M-MDSCs) (LY6G−/LY6Chigh), morphologically similar to monocytes, and polymorphonuclear (PMN) MDSCs (Ly6G+/LY6Clow) [164]. Hypoxia in the TME facilitates the expression of HIF-1alpha inducing the expression of CCL26 by tumor cells, leading to MDSC recruitment and accumulation [165]. PMN-MDSCs inhibit T cell functions via the production of reactive oxygen and nitrogen species, inducing T cell apoptosis or depletion. M-MDSCs are considered to show even more suppressive functions via high expression of Arg1, driving T cell anergy by depleting arginine pools. In addition, MDSCs can secrete high levels of IL-10 and TGF-beta, IFN-gamma [166] and produce reactive nitrogen species, negatively affecting T cell recruitment and activation [167]. They also exhibit tumor-promoting functions independent of immune suppression, such as the promotion of metastasis and angiogenesis via the production of VEGF, basic fibroblast growth factor (bFGF), and matrix metalloproteinase 9 (MMP9) [168,169]. Some studies linked MDSC accumulation with an increase in TME VEGF concentration during disease progression [170]. Moreover, MDSCs promote metastasis by participating in the creation of the premetastatic niche to enhance engraftment by circulating tumor cells [171]. MDSC cells escort also tumor cells into the circulation and promote their extravasation into the tissues [172]. Studies in CRC patients also show that human MDSCs enhance CRC cell stemness [171], which accelerates faster cancer cells proliferation. Toor et al. showed that levels of tumor-infiltrating MDSC were increased in patients with high tumor budding and advanced stage of CRC suggesting their potential roles in metastasis [173].

##### GM-CSF

In tumor microenvironment granulocyte-macrophage colony-stimulating factor (GM-CSF) is produced by MDSC cells, M2 macrophages, type 3 innate lymphoid cells (ILC3) cells, neoplastic colonic epithelial cells, T cells, mast cells, NK cells, endothelial cells, and fibroblasts [174], together with a wide variety of cancer cell types [175]. GM-CSF promotes the growth and migration of tumor cells by enhancing the expression of matrix metalloproteinases (MMPs) [176]. An increased level of GM-CSF in serum is considered a potential diagnostic and prognostic marker indicating poor prognosis in colorectal cancer patients [177,178]. Moreover, GM-CSF can decrease the apoptosis of colon cancer cells [179].

A large body of evidence supports the thesis that GM-CSF can act as a tumor-derived factor and may promote tumor growth and progression. GM-CSF induces autocrine and paracrine VEGF release by intestinal epithelial cells, promoting angiogenesis [180]. GM-CSF promotes the growth and migration of tumor cells by stimulation of MMPs expression [176]. Increased level of GM-CSF in serum is considered a potential diagnostic and prognostic marker associated with poor prognosis in CRC patients [177]. These results might suggest that GM-CSF in addition to immune-stimulatory functions may have direct effects on tumor progression and invasion [181]. Although the prevalent number of reports showed pro-tumor effects of GM-CSF [46], some studies suggested that GM-CSF has inhibitory effects on tumor growth and metastasis. It was shown, that GM-CSF can act on dendritic cells to promote their antitumor response [182] and on monocytes/macrophages to inhibit CRC cell proliferation [183]. GM-CSF also stimulated dendritic cell maturation and could augment *adenomatous polyposis coli* (*APC*) gene function [46].

In analyzed studies, GM-CSF was neither increased in patients staged I-IV nor I-III than in controls (Table 1). Notwithstanding, other studies (Table 1) show increased GM-CSF levels in each clinical-stage vs. controls, without differences across the subsequent stages. Therefore the role of GM-CSF in the development of CRC remains uncertain.

#### 2.1.5. Neutrophils N2 and Their Cytokines Panel

Tumor-associated neutrophils (TANs) play a key role at each stage of CRC, in tumor initiation, progression, and metastasis [184]. After stimulation neutrophils acquire the ability to polarize to antitumor (N1) or pro-tumor (N2) phenotype in TME. N1 neutrophils have features of classical neutrophils, whereas N2 phenotype neutrophils show typical features of PMN-MDSCs [164]. Pro-tumor N2 neutrophils induce progression of the disease and release of CXCL1, MMP9, VEGF, and TNF-alpha [185]. CRC tumor microenvironment stimulates neutrophil plasticity via cytokines and growth factors [186]. It was shown, that neutrophil plasticity and localization at the tumor site depends on the type and the stage of the tumor [187].

N2 neutrophils act via producing and releasing ROS and nitric oxide (NO), which increase DNA instability. Pro-tumor N2 TANs can inhibit T cell proliferation via expression of arginase 1 and induce T cell apoptosis via NO production [188]. N2 TANs could contribute to tumor invasion and angiogenesis through the production of MMP9 and VEGF in the primary and metastatic sites [12].

It was found that TANs from early tumors showed more cytotoxic properties toward cancer cells and produced higher levels of TNF-alpha, NO, and H2O2 showing antitumor N1 features. In established tumors, these functions were down-regulated and TAN acquired pro-tumorigenic phenotype [189]. It might suggest that neutrophils phenotype is more dependent on TME than of other cells. The high number of intratumoral neutrophils was reported to be associated with unfavorable recurrence-free, cancer-specific, and overall survival [190]. On the contrary, it was documented that neutrophil infiltration was a favorable prognostic factor for the early stages (I-II) of CRC [191], and patients with high tumor infiltration with CD177+ neutrophils had better overall survival (OS) and disease free survival (DFS) [192]. It could be assumed, that N2 neutrophils present in the tumor in later stages could contribute to increased levels of VEGF, IL-8, and TNF-alpha in advanced stages CRC patients. Early stages CRC tumors neutrophils seem to exhibit N1 phenotype.

##### TNF-Alpha

Most studies suggest that TNF plays mainly the pro-tumor role in CRC development. It was found, that TNF expression is increased in CRC tissues and TNF serum levels correlate with CRC progression and reduced patient survival [193]. Moreover, TNF-alpha signaling drives the accumulation of MDSCs by promoting their survival [194]. Activation of the signaling cascade of the TNF receptors can result in the nuclear translocation of NF-kappa beta and activator protein 1 (AP-1), which promotes cell survival, proliferation, angiogenesis, tumor promotion, and metastasis [195,196]. The binding of TNF to tumor necrosis factor receptor 2 (TNFR2) triggered the proliferation of CRC cell lines in a STAT3-dependent manner [197], similarly to the IL-6 activation.

TNF-alpha activates NF- kappa beta signaling, thereby contributing to inflammation, cell survival, proliferation, The transcription factor NF- kappa beta links inflammatory signaling and cancer. NF- kappa beta promotes tumor metastasis by regulating epithelial-mesenchymal transition in CRC [198]. Few reports indicated that TNF-alpha could also have an antitumor effect in CRC. The net contribution of TNF to CRC may be determined by the timing of its secretion during tumorigenesis or the type of immune cells secreting it [46]. Activated M2 macrophages, but also MDSC cells are considered to be the main producers of TNF-alpha in CRC [199,200].

TNF-alpha was found elevated in early-stage I patients compared to controls. In the later stages II, III, and IV, TNF-alpha continued to be increased (Table 1). However, TNF-alpha levels were not increased in stage IV patients compared to earlier stages (I-III) (Table 2). This finding may suggest a more pronounced role of TNF-alpha in earlier stages of CRC, with the contribution of MDSC and M2 macrophages to its increase.

#### 2.1.6. Tregs Lymphocytes and Their Cytokines Panel

Treg lymphocytes are considered to play a major role in creating immunosuppression within TME, contributing to cancer immune escape mechanisms and tumor growth [201]. Tregs can suppress the function of cytotoxic T cells and antigen-presenting cells by cell-cell interactions as well as suppress cytokines release including IL-10, TGF-beta, and interleukin 35 (IL-35) in CRC TME [202].

A decreasing number of Tregs with tumor stage in CRC was shown by Reimers et al., who demonstrated Foxp3+ cells above-median were more prevalent in stage I tumors [203]. Knowing that early stages CRC tumors contain a higher number of Tregs cells [7], we could hypothesize that Tregs are the source of TGF-beta and IL-10 in less advanced stages. Several studies confirmed, that M2 macrophages and MDSC cells infiltration level increases with the tumor growth and tumor stage [203,204]. Therefore, M2 TAMs and MDSC cells could contribute to the elevated levels of IL-10 and TGF-beta in the advanced CRC stage.

#### 2.1.7. Tumor Cells and Their Cytokines Panel

CRC cells express highly variable amounts of different cytokines, chemokines, and growth factors in vitro [205]. These include IL-6, interleukin 8 (IL-8), CCL2, macrophage colony-stimulating factor (M-CSF), GM-CSF, CXCL10, CXCL12, VEGF-A but also granulocyte colony-stimulating factor (G-CSF) [206,207]. These molecules contribute to a variety of functions related to systemic inflammation and cancer progression. IL-8 is an important proinflammatory chemokine, recruiting granulocytes and also promoting angiogenesis [208]. Both M-CSF and GM-CSF stimulate the proliferation, differentiation, and survival of monocytes and macrophages. M-CSF plays a role in M2-like anti-inflammatory macrophage polarization and GM-CSF contributes to M1-like proinflammatory macrophage polarization [209].

##### G-CSF

G-CSF can be produced by colon tumor cells. G-CSF was also found to be highly produced by stromal myofibroblasts and carcinoma cells [210]. G-CSF has a direct effect on tumor cells promoting tumor stem cell longevity, their proliferation, and migration. In addition, G-CSF may promote pro-tumorigenic immune cell phenotypes such as M2 macrophages, myeloid-derived suppressor cells, and regulatory T cells [211].

Of note, the G-CSF receptor (G-CSFR) is highly expressed in 90% of human gastric and colon carcinomas [210]. Moreover, exposure of carcinoma cells to G-CSF led to increased proliferation, migration, and expansion of a sub-population of carcinoma cells expressing stem-like markers. These processes are dependent on extracellular signal-regulated protein kinase 1/2 (ERK1/2) and ribosomal S6 kinase 1 (RSK1) phosphorylation. These data suggest that the G-CSF/G-CSFR axis promotes colorectal cancer development and suggest that they are potential tumor targets [210]. The highest expression of ligand/receptor was demonstrated in T3 stage tumors, suggesting that G-CSF might contribute to cancer development via stimulation of tumor cell migration. High levels of G-CSF could accelerate proliferation enhancing tumor heterogeneity whereas intensified migration could result in initiating tumor cell mobilization needed for metastasis [210].

Increased G-CSF level was shown in the joined group of CRC patients staged I-IV or I-III than in controls. When patients’ stages were assessed separately vs. control, G-CSF level was not elevated in stage I patients, but was increased in separately assessed stages II, III, and IV patients (Table 1). When comparing G-CSF levels among clinical stages, the difference was significant for stage IV compared to stage I, only (Table 2).

The dynamics of G-CSF increase suggests this factor may play a particularly important role in the later stage of CRC development. Thanks to its multifactorial action, G-CSF could contribute to tumor cell longevity and immune-suppressive TME in CRC. In early tumors, G-CSF could be produced mainly by M2 macrophages and MDSC. At later stages, due to the increasing tumor size, cancer cells could contribute to the increased level of G-CSF.

### 2.2. Antitumor Cells and Their Cytokines Panel

#### 2.2.1. Lymphocytes Th1

Th1 lymphocytes support the cytotoxic action of CD8 T lymphocytes, which serve as the main cell defense against tumor cells. Th1 helper cells enhance cell-mediated response, mediated by macrophages and cytotoxic T cells [212]. The primary effector cells of Th1 immunity are macrophages, CD8 T cells, IgG B cells, and IFN-gamma CD4 T cells. Th1 cells secrete IL-2, IL-12, TNF-alpha, IFN-gamma. The majority of them including IFN-gamma, interleukin 2 (IL-2) and interleukin 12 (IL-12) were shown to exert an antitumor effect [213].

##### IL-2

IL-2 is considered an important antitumor cytokine. IL-2 activates NK cells and T cells. The major challenge in the development of IL-2 as a therapeutic antitumor agent is that IL-2 can act on both T cells and Tregs [214]. IL-2 also stimulates effector T cells and differentiation of naive CD8+ T cells into memory T cells [215]. Together with other cytokines, IL-2 supports naive CD4+ T cell differentiation into Th1 and Th2 lymphocytes but it inhibits differentiation into Th17 and follicular Th lymphocytes [216,217]. IL-2 plays a key role in enduring cell-mediated immunity [218].

The major sources of IL-2 are activated CD4+ T cells, activated CD8+ T cells, and DC cells [219]. Disease progression and negative prognosis in cancer are associated with reduced IL-2 concentrations or an increase in soluble IL-2 receptor concentrations [220]. Lack of IL-2 increase in the majority of presented studies (Table 1) both in early and metastatic stages may indicate the prevalence of pro-tumor processes in analyzed CRC groups.

##### IL-12

IL-12 plays a central role both in the induction and the expansion of Th1 responses as well as the activation of cytotoxic cells, like NK and CD8+T cells [221]. IL-12 activates and induces IFN-gamma production in these cells, which limits tumor growth and formation of metastasis [222].

IL-12 is constituted of two subunits, IL-12p35 and IL-12p40; these subunits may form an agonistic IL-12p70 heterodimer or an antagonistic IL-12p80 homodimer [223]. The IL-12p35 subunit is shared to generate IL-35 [224], whereas IL-12p40 is shared to form IL-23 [225]. Therefore, it is difficult to assess the effect of IL-12 without interfering at the same time with IL-23 or IL-35 signaling. In CRC patients high preoperative IL-12p40 serum levels predicted a longer survival [226].

In the intestine, dendritic cells, macrophages, and B cells have been reported to produce IL-12p35 and IL-12p40. Their stimulation by lipopolysaccharides and IL-10 is necessary for IL-12 production [227].

In analyzed studies, IL-12p40 was increased in all CRC patients with the highest level in stage I in comparison to more advanced stages (Table 2). This might be a sign of predominance of antitumor activity in the early stages of CRC TME. This hypothesis is in accordance with high NK/Th1 infiltration data in the early CRC stages.

In other studies, IL-12 was not increased in CRC patients staged I-III, I-IV, or stage IV than in controls, which could be explained by shifting the immune response into tumor-promoting Th2 cells. However further studies would be necessary to explain this observation.

##### IFN-Gamma

IFN-gamma stimulates antitumor immunity [228]. IFN-gamma signaling is a key factor for the polarization of Th1 immune responses. It enhances MHC class I antigen representation and promotes cytotoxicity of CD8+ T cells, NK cells, and macrophages. Polarization of Th1 response enhanced by IFN-gamma correlates with prolonged survival of CRC patients [229]. There is an association between high serum IFN-gamma and the absence of nodal metastases in CRC patients [2]. The data suggest that IFN-gamma induces a protective, antitumor response in CRC patients. However, it was also demonstrated that IFN-gamma could increase intestinal permeability which might increase intestinal inflammation and stimulate CRC formation [230]. Moreover, IFN-gamma can activate inducible nitric oxide synthase (eNOS) to produce nitric oxide free radicals.

IFN-gamma is known to be produced predominantly by NK and NKT cells, also by CD4 Th1 cells and CD8 cytotoxic T lymphocyte (CTL) after their activation [231]. Moreover, also non-cytotoxic ILC3 cells, M1 macrophages, and mucosal epithelial cells IFN-gamma produce this cytokine [232].

In analyzed clinical studies, IFN-gamma was elevated in patients staged I-III and I-IV in relation to controls, and in stage IV patients compared to less advanced stages (I, II, III), confirming the importance of this cytokine in CRC development (Table 1 and Table 2). The source of IFN-gamma in early tumors could be Th1 lymphocytes and cytotoxic CD8 cells together with NK cells, while in the advanced stage the source of IFN-gamma could probably be M1 macrophages stimulated by IL-12 or IL-18 [233].

#### 2.2.2. M1 Macrophages and Their Cytokine Panel

M1 macrophages phenotype support antitumor immunity and secrete IL-12, IL-6, TNF-alpha, IL-23, interferon-alpha (IFN-alpha), interferon-beta (IFN-beta), INF-gamma, interleukin 36 (IL-36), and interleukin 15 (IL-15). Several factors contribute to the M1 polarization including IFN-gamma, TNF-alpha, also pathogen-associated molecular patterns (PAMPs) and damage-associated molecular patterns (DAMPs), heat shock proteins. M1 polarized macrophages produce large quantities of pro-inflammatory cytokines including TNF-alpha, IL-1 beta, IL-6. M1 macrophages promote immune responses by up-regulation of MHC-II, in conjunction with co-stimulatory molecules, Th1- cells, Th17-derived cytokines, and chemokines [234].

Ong et al. reported that M1 TAMs were pro-inflammatory and inhibited the proliferation of tumor cells. M1 TAMs produced cytokines (e.g., IL-6 and IFN-gamma) and chemokines (e.g., IL-8 and CCL2) that attract T cells, promoting type-1 T cell responses. Using CRC tissues, the authors confirmed that pro-inflammatory TAMs in vivo correlated with the number of tumor-infiltrating T cells. TAMs induced antitumor effects with the help of T cells [235]. Another study conducted on close to 500 CRC specimens showed a parallel infiltration of M1 and M2 cells at the tumor front, with an inverse correlation of both phenotypes with tumor stage. No difference was detected in cancer-specific survival (CSS) in CRC groups with different M1/M2 macrophage ratios. However, the studies could suggest that the presence of numerous M1 macrophages could be favorable in patients with CRC, despite the presence of M2 macrophages [11]. The presence of M1 macrophages in CRC tumors and positive correlation with the presence of T cells contributes to a picture of “inflamed TME” and may be correlated with a better prognosis.

##### IL-23

Dendritic cells, macrophages, and neutrophils were shown to produce IL-23 during intestinal inflammation [236,237]. The evidence suggests that IL-23 may indirectly promote tumor cell survival. Namely, IL-23 has been reported to drive intestinal inflammation by inducing other pro-inflammatory cytokines secretion, such as IL-6, IL-17, and IL-22 [238]. These cytokines might activate tumor cell proliferation through STAT3 and NF-kappa beta pathways. A variety of hematopoietic cells in the intestine may react to IL-23, including ILCs, Treg, and Th17 cells. The biological effect of IL-23 signaling may be heterogeneous in different cell populations. IL-23 signaling promotes IL-22 secretion by ILCs and IL-17 production by Th17 cells, while Treg cell activation is inhibited [239].

In the serum of CRC patients, IL-23 levels were reported to be increased [240]. IL-23 was increased in patients staged I-IV vs. control, and also in patients staged I, II, III, and stage IV compared to controls (Table 1). IL-23 was elevated in CRC patients III/IV vs. I/II and stage IV patients vs. I-III (Table 2), suggesting a more prominent role in advanced CRC stages. Other authors reported stable levels of IL-23 with no significant differences between disease stages [31]. The role of IL-23 seems to be uncertain at this stage of the investigation.

##### IL-15

IL-15 is produced by a range of cells, including stromal cells, epithelial cells, and myeloid cells such as monocytes, macrophages, and dendritic cells. Dendritic cells, cytotoxic CD8+ T cells, and NK cells all express the IL-15 receptor that consists of three distinct receptor chains [241,242]. Cytotoxic T and NK cells represent the most important immune effectors to integrate the antitumorigenic function of IL-15 by activating the APO-1/FAS- or granule-mediated cytotoxic pathway [243]. Thus, IL-15 regulates antitumor cytotoxicity and modulates the inflammatory tumor microenvironment. Moreover, IL-15 is expressed in human CRC cells [244]. IL-15 maintained homeostasis and induced activation of NK cells and CD8+ memory T cells [245]. IL-15 is negatively involved in CRC progression via inhibiting the proliferation and promoting apoptosis of CRC cells. Moreover, it was demonstrated that IL-15 exhibited an antitumor effect via inhibiting the proliferation and promoting apoptosis of CRC cells. Of note, overexpression of IL-15 caused by gene vector was shown to reduce angiogenesis in CRC, which further suggested the positive effect of IL-15 against the invasion and metastatic spreading of CRC cells [246].

Serum IL-15 level was neither increased in early nor advanced CRC stages. This fact could be a reflection of pro-tumor TME processes in analyzed populations, however, requires further work.

#### 2.2.3. Innate Lymphoid Cells (ILCs) and Their Cytokine Panel

Innate lymphoid cells are divided into five subpopulations: NK cells, innate lymphoid cells type 1 (ILC1), innate lymphoid cells type 2 (ILC2), ILC3, and lymphoid tissue-inducer (LTi) cells based on their differentiation, transcription factors, and cytokine expression [247]. The role of ILC cells seems to be heterogeneous in CRC, with NK and IL2C cells required for antitumor immunity [57], and ILC1, ILC3 cells supporting the development of CRC [248].

##### NK Cells

It is acknowledged that NK cells are key antitumor primary effectors to eliminate CRC cells without prior immunization, and their altered phenotype or dysfunction in CRC patients can result in the limitation of the immune response, associated with the lower survival rate [46]. NK cells can effectively induce cancer cell death, including CRC stem cells and cancer-initiating cells [249]. NK cells apart from their cytotoxic activity can produce IFN-gamma, TNF-alpha, GM-CSF, and IL-8 [250]. De Vries et al. showed that NK cells (identified as CD127−CD56+CD45RO+) were prevalent among the CRC ILC cells population [57].

##### ILC1

ILC1 and NK cells are mainly engaged in the protection against viruses, bacteria, and cancer [251] through the secretion of IFN-gamma and GM-CSF [250]. ILC1s are characterized by the secretion of proinflammatory cytokines like IFN-gamma and TNF-alpha [252].

##### ILC2

It is known that ILC2 cells secrete type-2 cytokines including IL-4, IL-5, IL-13, and GM-CSF [253]. IL-13 signaling in CRC has been associated with a poor prognosis [254], ILC2-derived IL-13 activates MDSCs, which promote pro-tumor TME response [51]. High levels of the ILC2-derived IL-33 and IL-4 were associated with poor prognosis [52]. However, IL-5 secreted by ILC2 is important for the development, recruitment, activation, and survival of eosinophils [255] associated with antitumor response and good CRC prognosis [256]. It was also shown that the ILC2 cytokines, IL-5, and GM-CSF could control tumor growth-as genetic ablation of IL-5 and GM-CSF increased the tumor burden in the murine CRC model [257]. Eosinophils were shown to protect against tumor development via IL-5 and GM-CSF driving their migration to the tumor and promoting antitumor Th1 cell responses [257]. Moreover, ILC2 cells have been shown to promote DC migration and cytotoxic T cell activation, which support antitumor immunity through the secretion of IL-13 [38,52]. The antitumor role of ICL2 is supported by the finding ILC2 signature was associated with increased overall survival in CRC [258]. However, the frequency of ILCs within the tumor microenvironment is low compared to other adaptive immune cells, which could diminish their role in cytokine production.

##### ILC3

ILC3 produces mainly IL-17 and IL-22, important for mucosal immune responses and tumor progression [259]. Moreover, the activity and function of ILC3s are regulated by IL-23 produced by activated intestinal dendritic cells and macrophages [225], shown to promote CRC development [260].

In the normal colon mucosa, ILC3s represented the most abundant subset, followed by ILC1s and ILC2s. In resected CRC tumor samples of stages II and III, there was a marked reduction in the number of ILCs. Moreover, ILC2 frequency was low in tumor tissues, while the number of ILC3s was decreased and the number of ILC1s was increased [57].

Immunohistochemical analysis of epithelial crypts confirmed decreased numbers of infiltrating ILC3s in the tumor tissues as compared with normal tissues. ILC3s are thought to play a protective role against cancer as suggested by some studies [261,262]. Thus, their reduction, together with the increase of ILC1s, could play a role in malignant transformation and tumor progression. Moreover, altered ILC1/ILC3 balance might be dependent on the plasticity of ILC3s driven by the CRC microenvironment, where cytokines including IL-1 beta, IL-15, and IL-12 could convert ILC3s into IFN-gamma producing ILC1-like cells [263].

Altogether, ILC cells including NK cells could contribute to the increased level of TNF-alpha, GM-CSF, IFN-gamma, IL-4, IL-5, IL-13, IL-17, and IL-22, modulating TME and contributing to tumor progression or antitumor activities. However, due to the low numbers of these cells in TME, the impact of these cells on cytokines levels is probably limited.

#### 2.2.4. Dendritic Cells

Dendritic cells are much less frequent in tumor TME, however, they play a key role in antitumor response. A simplified classification of DC types based on origin, gene expression, phenotype, functions, and localization was proposed [264]. The following types of DCs can be found: plasmacytoid DC (pDC), myeloid/conventional DC1 (cDC1), myeloid/conventional DC2 (cDC2), and monocyte-derived dendritic cells (MoDCs).

Myeloid cDC1 has been characterized as a subset of DC with a high intrinsic capacity to cross-present antigens via MHC class I to activate CD8+ T cells and to promote Th1 and NK cells responses through IL-12 [265,266,267]. In vitro, human cDC2 were shown to be potent activators of Th1, Th2, Th17, and CD8+ T cells [268] suggesting that possibly they would be able to promote immune responses in vivo.

Mo-DCs studied ex vivo secrete IL-1, TNF-alpha, IL-12, IL-23, stimulate CD4 and CD8 T cells and express CCR7 [269]. MoDCs do not migrate efficiently to lymph nodes and are particularly prone to develop immunosuppressive functions, whereas cDC1 excel in the activation of CTLs [270]. Plasmacytoid dendritic cells can secrete INF-alpha, IL-6, granzyme B; cDC1 cells produce IL-12, CXCL9, CXCL10, TNF-alpha, IFN-gamma, and cDC2—IL-1, IL-8, IL-10, IL-12, IL-23, and TNF-alpha [271].

Several studies reported a decreased number of DC in advanced CRC stages in comparison to early stages. They showed that patients with locally advanced tumors (T3–T4) had significantly lower infiltration with CD83+-matured DCs in the tumor stroma and invasive margin. The number and localization of tumor-infiltrating DCs and tumor-infiltrating lymphocytes (TILs) were decreasing with tumor progression [272,273].

It was demonstrated, that between colon adenomas and CRC, the density of mature DCs decreased, but the density of immature DCs was gradually increased [274]. Accumulation of immature dendritic cells could contribute to the suppression of dendritic cells and T cells by activation of indoleamine 2,3-dioxygenase and arginase 1 by tumor-derived growth factors. This could lead to the induction of polarized CD4+Th 2 cells promoting the expansion of cancer cells at the expense of CD8+T cells [275].

In several studies, higher cDC1 infiltration in tumors was correlated with higher infiltrations of CTLs or NK cells, suggesting their cooperation in the antitumor response [276,277]. Based on analyzed studies, we suggest that dendritic cells could contribute to increased levels of cytokines, especially in the early stages of CRC.

#### 2.2.5. Neutrophils N1

N1 TANs exert antitumor activity. N1 phenotype of TANs, activated by type I interferons, inhibits angiogenesis and effectively eliminates tumor cells via antibody-dependent cellular cytotoxicity (ADCC) and phagocytosis. Neutrophils recruit and activate immune cells by producing cytokines, chemokines, and proteases and can stimulate T cells proliferation as well as NK and DC cells maturation [278]. N1 TANs show increased NADPH oxidase activity which leads to the production of reactive oxygen species, cytotoxic to cancer cells [279].

The immune profile of N1 TANs is characterized by secreting high levels of TNF-alpha, CCL3, intercellular adhesion molecule 1 (ICAM-1), and low levels of arginase. The N1 neutrophils are short-living, highly cytotoxic cells and show a mature phenotype with high immune-stimulating activity. Contrary, N2 neutrophils, are long-living, have low cytotoxic properties, show an immature phenotype, and have a highly pro-angiogenic, pro-metastatic, and immunosuppressive activity. The heterogeneity of TANs, their plasticity, and their dual function in tumors are regulated by several TME factors and signals, mainly TGF-beta and IFN-beta signaling [280]. The infiltration of the tumor by neutrophils showed a relatively low abundance, compared to macrophages M2 and Tregs in CRC [184].

It was demonstrated that a decreased number of intratumoral neutrophils correlates with metastasis/tumor size (pM/pT) status, clinical stage, and shorter survival in CRC patients, being an independent poor prognostic factor [281]. On the contrary, CRC tissues from patients with well-to moderate tumor differentiation, fewer metastases, TNM stage I or II diseases, or rectum cancer showed higher TAN abundances and lower Treg or TAM abundances [282], suggesting their crucial role in shaping the response against the tumor. High infiltration of tumors with N1 TANs could contribute to increased levels of TNF-alpha in the circulation.

#### 2.2.6. B Cells

B cells can regulate immune responses via humoral mechanisms, including inhibition of T cell responses [283]. B cells are divided into cytokine-producing ‘regulatory’ and ‘effector’ B subsets. Regulatory B cells secrete IL-10 and TGF beta-1, while effector B cell populations produce IL-2, IL-4, TNF-alpha, IL-6 (Be-2 cells), or IFN-gamma, IL-12, and TNF-alpha (Be-1 cells) [284].

In patients with advanced CRC stages, significantly lower levels of tumor-infiltrating B cells were described [285]. The B-cell infiltrates of CRC tumors were characterized by the accumulation of differentiated memory B cells or plasma cells. Moreover, advanced tumors and metastases were infiltrated by a considerable number of regulatory B cells. Also, regulatory B cell subsets (CD24highCD38high) were significantly elevated in advanced CRC tumors samples infiltrates [286]. These cells were identified as the major B cell population secreting IL-10 in humans [287]. Therefore, Breg cells might contribute to the increased level of IL-10 in colon cancer TME.

#### 2.2.7. Cytotoxic T Cells

Secretion of perforins and granzymes is the main way of cytotoxic CD8 T cells activity. Most cytotoxic CD8 T cells also release IFN-gamma, TNF-alpha, and tumor necrosis factor-beta (TNF-beta). IFN-gamma induces expression of MHC class I. IFN-gamma activates macrophages, recruiting them to sites of infection both as effector cells and as antigen-presenting cells [288]. Contrary, CD8+ T cells were found at lower levels in CRC patients with the advanced-stage disease [285]. Infiltration of invasive margin and tumor core by CD3CD8 lymphocytes resulted in longer DFS in stages II and III and improved OS in stage III. This confirms their positive prognostic value in CRC [289]. Despite the mainly cytotoxic activity of CD8 cells, we presume they could contribute to some extent to increased levels of IFN-gamma, TNF-alpha, and TNF-beta in CRC patients.

## 3. Inflammation in Colon Cancer Development

CRC develops in a sequence of histological, morphological, and genetic changes. Sporadic and colitis-associated colon cancer (CAC) develop in different ways. Sporadic CRC develops in an adenoma-carcinoma sequence, with inflammation following tumor development [3,290], whereas in colitis-associated cancer CRC is induced by inflammation-induced dysplasia.

Adenoma-carcinoma sequence is responsible for the majority of CRC cases. In this case, tumor-initiating event (environmental mutagens, spontaneous mutations) leads to the accumulation of mutations or epigenetic alterations in the intestinal epithelial cells. This can cause uncontrolled proliferation of abnormal cells, promoted by cytokines and growth factors secreted by infiltrating cells, mainly macrophages and neutrophils [291]. The excessive growth of clonal cells contributes to aberrant crypt foci formation, development of early adenomas, late adenomas, and finally the CRC tumor [292]. Inflammatory cells actively contribute to the mucosal immune response. It was shown that number of macrophages and neutrophils was increasing from low to high-grade dysplasia polyps and was highest in invasive adenocarcinoma samples [291]. A recent study showed Th2 and Th17 cells immune pathways were activated in normal to adenoma transition, whereas Treg cells were activated in adenoma to carcinoma transition [293]. Elevated levels of reactive oxygen and nitrogen species secreted by neutrophils and macrophages may be associated with further DNA injury [294]. Mutations in intestinal epithelial cells can affect the regulation of COX-2 (cyclooxygenase-2) expression. COX-2 was found to be overexpressed in colonic polyps compared with paired adjacent normal mucosa [295], and in CRC tissues, suggesting a positive role of COX-2 in early colorectal carcinogenesis. Pro-tumor action of COX-2 is supposed to be exerted via generating prostaglandin E2 (PGE2), enhanced angiogenesis, cell survival, and activation of the WNT pathway [296,297].

Cancer-associated colitis may be associated with inflammatory bowel disease (IBD), infections, or abnormal immune reactions. IBD (ulcerative colitis and Crohn’s disease) is responsible for approximately 2% of CRC cases [298]. Active inflammation in IBD patients results in increased numbers of inflammatory cells at the beginning of the process. Neutrophils and macrophages produce free radicals and reactive nitrogen forms [299], which can damage genes preventing carcinogenesis (including *p53*, DNA mismatch repair proteins), transcription factors (nuclear factor-kappa beta), or signaling proteins such as cyclooxygenases [300]. These changes together with chronic inflammation can lead to excessive tissue regeneration, proliferation, and clonal expansion of initiated tumor cells (tumor promotion) as well as acquiring stem-like properties by previously differentiated cells [292]. This can result in uncontrolled growth and tumor formation.

Some authors underline the differences in the timing of genetic alterations between sporadic and colitis-associated cancers. The development of sporadic cancer includes among others loss of *APC* gene function, aneuploidy, methylation changes, microsatellite instability, activation of *KRAS* gene, COX-2 enzyme, and loss of *p53* gene function [299]. In IDB-associated cancer loss of *APC* is less frequent and happens later in the tumor development, whereas loss of *p53* function occurs earlier and is more frequent in IBD-associated cancer, compared to patients with sporadic CRC [299,300,301].

## 4. Conclusions

This review is an attempt at cytokine kinetics analysis in CRC tumors. The analysis recapitulates an existing hypothesis that cytokine profile depends on the TNM stage [2,302]. It was observed, that in CRC TME both the immunostimulation and immunosuppression take place, with more pronounced immune suppression in advanced stages [57]. Inflammation co-exists and contributes to cancer development and progression [2,303]. Cytokines profile may provide data of the immune status of TME at each stage: ’cold’/’frozen’ or ‘hot’, contributing to patients’ prognosis analysis. The cytokine profile reflects numerous processes, including angiogenesis, lymphangiogenesis [304], increased systemic inflammatory response, and acquisition of a stem-like phenotype by cancer cells [290,305], factors facilitating metastasis. This way cytokines profile assessment could possibly contribute to the choice of more personalized treatment. Larger studies are necessary to establish the roles of cytokines for different CRC stages and the importance of their kinetics to patients’ prognoses.

## 5. Limitations

We acknowledge several limitations of the presented review. First, sources of the circulating cytokines are difficult to be established, which requires further studies. Moreover, analyzed studies consisted of heterogeneous patient groups: before and after surgery, with and without metastases. Reported cytokines levels were not consistent among studies, which could reflect various methods of their assessment, small analyzed cohorts, and patients’ heterogeneity. Only a few cytokines were discussed, and chemokines were not included due to the space limitations. Th9 cells were not discussed in this review due to limited and contradictory reports regarding the role of these cells in CRC in the literature. Th22 cells were not included in the discussion due to limited clinical data of its main cytokine IL-22 in respective CRC stages. In the presented review some important factors were not taken into account, including microsatellite instability (MSI) status, chromosomal instability (CIN) status–both may significantly impact tumor TIL infiltration [306]. Other important factors include CpG island methylator phenotype status, *BRAF* and *KRAS* mutational status, tumor differentiation (grade), location (right-sided vs. left-sided) [307], and colon microbiota. These factors will be included in future reviews.

## Data Availability

Not applicable.
